# Time of flight secondary ion mass spectrometry imaging of contaminant species in chemical vapour deposited graphene on copper

**DOI:** 10.3762/bjnano.17.13

**Published:** 2026-01-21

**Authors:** Barry Brennan, Vlad-Petru Veigang-Radulescu, Philipp Braeuninger-Weimer, Stephan Hofmann, Andrew J Pollard

**Affiliations:** 1 Atlantic Technological University, Ash Lane, Sligo, F91 YW50, Ireland; 2 National Physical Laboratory, Hampton Road, Teddington, TW11 0LW, United Kingdom; 3 Department of Engineering, University of Cambridge, Cambridge CB3 0FA, United Kingdom

**Keywords:** contamination, copper, CVD, graphene, ToF-SIMS

## Abstract

Time of flight secondary ion mass spectrometry (ToF-SIMS) was used to probe the chemistry of graphene grown on copper foil substrates by chemical vapour deposition (CVD) under various growth conditions. The surface sensitivity, mass resolving power, and imaging capability of ToF-SIMS allow us to explore variations in the chemical species present on the graphene surface, as well as in three dimensions under the graphene. In this way, we can observe the impact that variations in the chemical composition of the copper foil have on the growth of the graphene; in particular, the accumulation of contaminations present in the copper foil, which has implications for the potential electrical properties of the graphene. We also observe variations in the permeation of oxygen underneath the graphene layers, resulting in oxidation of the copper substrate, depending on processing conditions employed and the chemical species present on the surface. This has implications for the gas permeation barrier properties of this material, graphene transfer mechanisms, as well as the effectiveness of using the oxidation of the copper foil as a rapid graphene quality control method. These results highlight the significance of understanding the role of trace contaminants and elemental distributions within the catalyst in conjunction with growth parameters for optimised CVD of graphene layers.

## Introduction

The development of high quality, high throughput, and highly consistent chemical vapour deposition (CVD) processes for the growth of graphene is one of the major milestones that need to be overcome before the potential properties of graphene can be fully realised for device purposes [1–4]. While nanocomposites incorporating graphene are expected to be disruptive in their own right [5–6], it is the potential for large area, single crystal graphene sheets, which CVD growth can realise [7–10] which may have the greatest impact. Such sheets are expected to harness the full potential of the material and lead to a step change in terms of high speed [11–13], transparent [14] and flexible electronic devices [15], membranes [16–17], and sensors [18–19]. A large amount of work has been dedicated to the optimisation of the growth process to aid in the formation of large area single crystals [3,20–23], utilising Raman spectroscopy to confirm the physical structure of the graphene [24–26] and X-ray photoelectron spectroscopy (XPS) to confirm the sp^2^ bonding configuration [27–28]. However, there is typically little consideration given to possible chemical contaminants in as-grown graphene [29] or contamination associated with processing [30–33]. Trace contaminants can significantly influence the catalytic growth process, as well as the post-growth processing required for electronics, and can be deleterious for many applications. There is a need for characterisation techniques with the ability to distinguish different chemical species present, combined with surface sensitivity and suitably high spatial resolution.

We here focus on time-of-flight secondary ion mass spectrometry (ToF-SIMS) imaging of Cu-catalysed graphene CVD samples. ToF-SIMS offers high surface sensitivity (<1 nm depth), low detection limits (ppm and better), high mass resolution to aid identification of chemical species in both molecular and elemental forms [28–29,34], combined with below 30 nm lateral resolution possible under optimal conditions [35]. We investigate a range of different growth conditions and probe the surface of the graphene as well as the distribution of chemical species in three dimensions within the Cu foil [34]. Various chemical species are detected on the Cu surface, particularly phosphorous-, chlorine-, sulphur- and nitrogen-containing species, through diffusion of material present in the copper foil before growth [36–37], which could lead to variations in the properties of graphene once transferred from the Cu foil to an alternative substrate. We also explore the gas permeation properties of the CVD graphene on Cu [38] by examining the oxygen detected directly under the graphene after post-growth exposure to atmosphere. This could have implications for better understanding transfer mechanisms that rely on oxidation of the Cu substrate [39–40], defect characterisation [41], or the heat dissipation ability of graphene on Cu [42].

## Methods

CVD graphene sheets were grown on Cu foils (Alfa Aesar (46365), thickness of 25 µm, with a metal purity specified by the manufacturer of 99.8%), which are commonly used for this purpose [43]. Cu foil purity as indicated by the manufacturer is usually referring to the bulk metal content and does not typically include elements such as carbon or oxygen. Surface carbon contamination on Cu foils, particularly for higher-purity Cu foils, typically originates from the cold rolling process, where oils are important to balance friction properties and cool the strip and rolls. Surface roughness of the Cu foils was measured with a Wyko NT1100 White Light Optical Profiling System using a 20× magnification in vertical scanning interferometry (VSI) mode. Scanning electron microscopy (SEM) was carried out with a Carl Zeiss SIGMA VP at an acceleration voltage of 2 kV to ensure sample consistency.

Four separate Cu foil samples were prepared as described in detail previously [43]. For two of the samples, (labelled Ar and Ar:H_2_) the as-received Cu foils were used without any pre-treatment. The other two samples (labelled EP and BO) underwent two different pre-treatments, namely, either an electro-polishing step intended to remove the top surface layer of the foil and reduce surface roughness (EP) or a wet back side oxidation process to introduce oxygen into the Cu foil prior to growth (BO). The electro-polishing solution was prepared by mixing H_3_PO_4_ (85 wt % in H_2_O, Sigma Aldrich) in a 7:3 ratio with DI water. The cathode (Cu foil) area was chosen to be four times larger than the anode. The distance between cathode and anode was 4 cm. After electro-polishing the Cu foil was rinsed in a water jet for 5 min and then dried with N_2_ after dipping in isopropyl alcohol (IPA). The oxidation of the Cu foil was performed in a 30% H_2_O_2_ solution (Fisher Scientific) heated to 100 °C for 300 s. The Cu foil was gently placed on the H_2_O_2_ solution such that the Cu foil floated, and the top side was not exposed to hydrogen peroxide. Subsequently, the Cu foil was rinsed in DI water and IPA and dried with flowing N_2_. The surface roughness, measured over an area of 230 µm × 300 µm, from the BO, Ar, and Ar:H_2_ samples all had *R*_a_ values of ≈300 nm, with the EP value decreasing to ≈200 nm [43].

All graphene growth experiments were carried out in a commercial Aixtron Black Magic Pro 4-inch cold wall CVD system at a base pressure of ≈1 × 10^−2^ mbar. The Cu foils were placed into the CVD chamber and the temperature was ramped to 1065 °C at 100 °C/min in either an Ar atmosphere (Ar) or a 4:1 (200 SCCM:50 SCCM) mixture of Ar and H_2_ (Ar:H_2_). The samples were then held at 1065 °C for an annealing time of 30 min. Subsequently, the carbon precursor was introduced for 30 min. The graphene growth atmosphere consisted of 250 SCCM Ar, 26 SCCM H_2_ and 9 SCCM CH_4_ (0.1% diluted in Ar) for all samples except the BO sample where a flow rate of 30 SCCM CH_4_ (0.1% diluted in Ar) was used due to the very low nucleation density. After the growth step the reactor cooled to room temperature in an Ar only atmosphere. During all stages of the process, a pressure of 50 mbar was regulated via a PID controlled outlet valve.

Ex situ ToF-SIMS measurements were performed using a TOF SIMS IV instrument (ION-TOF Gmbh, Germany) at a vacuum pressure of <5 × 10^−9^ mbar. For 3D imaging, each depth profile was acquired by cyclically analysing a 150 µm × 150 µm area (with a pixel density of 128 × 128) from the centre of a 400 µm × 400 µm sputtered region during depth profiling, to mitigate crater edge effects on the generated spectra. 10 keV Cs^+^ ions with an ion current of 30 nA were used for sputtering cycles. The interleaved image spectra were acquired using 25 keV Bi_3_^+^ ions from a liquid metal ion gun, orientated at 45° to the sample surface. This was operated at an ion current of 0.1 pA, in an interlaced mode with a cycle time of 100 µs, in spectroscopy mode to give a mass resolving power (*M*/Δ*M*) greater than 5000. Origin of specific species were confirmed by further sample analysis using an OrbiSIMS instruments (Hybrid SIMS, IONTOF GmbH, Münster, Germany) incorporating an Orbitrap™ mass analyser (Thermo Fisher Scientific, Bremen, Germany) with a mass-resolving power of 240,000 at *m*/*z* 200. Depth profiles were acquired up to a depth of approximately 250 nm, with the thickness determined by acquiring reference profiles from a sample of known thickness, as well as comparing to the calculated sputter rate based on the expected sputter yield of Cu with 10 keV Cs^+^ ions. Due to the polycrystalline nature of the Cu foils, which can lead to variations in the sputter yield for different crystal orientations, the depth profiles were referenced relative to either the total ion intensity to provide a comparison of the oxygen distribution underneath the graphene layers, or normalised to the maximum ion intensity to highlight location of species at low concentration. To probe the surface of the samples in more detail, larger 500 µm × 500 µm surface images (with a pixel density of 256 × 256) were also acquired with 25 keV Bi_3_^+^ ions for longer acquisition times to enhance the ion signals. In some instances, multiple images were stitched together to provide imaging over larger areas. The ToF-SIMS images are presented as Red + Blue + Green overlays, with each colour representing a different ion species, and additive colour mixing; hence, where all three species are significantly present, a white signal is observed. Images from various locations on the samples were acquired to confirm the nature of the graphene coverage and variations in surface chemistry observed, with representative images shown in the figures. Measurements were acquired from samples that had been exposed to atmospheric conditions for up to four weeks before measurements, which facilitated oxidation of the surface of the Cu foils where no graphene was present.

In a manner analogous to previous work [44], XPS measurements were then carried out using an Axis-Ultra (Kratos Analytical, UK) operating at a pass energy of 40 eV for high resolution, narrow scan window spectra (100 meV step size, 500 ms dwell time), and 160 eV for wide scans (1000 meV step size, 200 ms dwell time), using a monochromated Al Kα X-ray source, with a photon energy of 1486.7 eV. Spectral peak fitting was carried out using CasaXPS (version 2.3.26PR1.0) with Shirley-type backgrounds for the high-resolution spectra, and elemental composition was calculated from the wide scans using the NPL transmission functions and average matrix relative sensitivity factors after removal of a Tougaard or linear background [45].

## Results and Discussion

**Figure 1:**
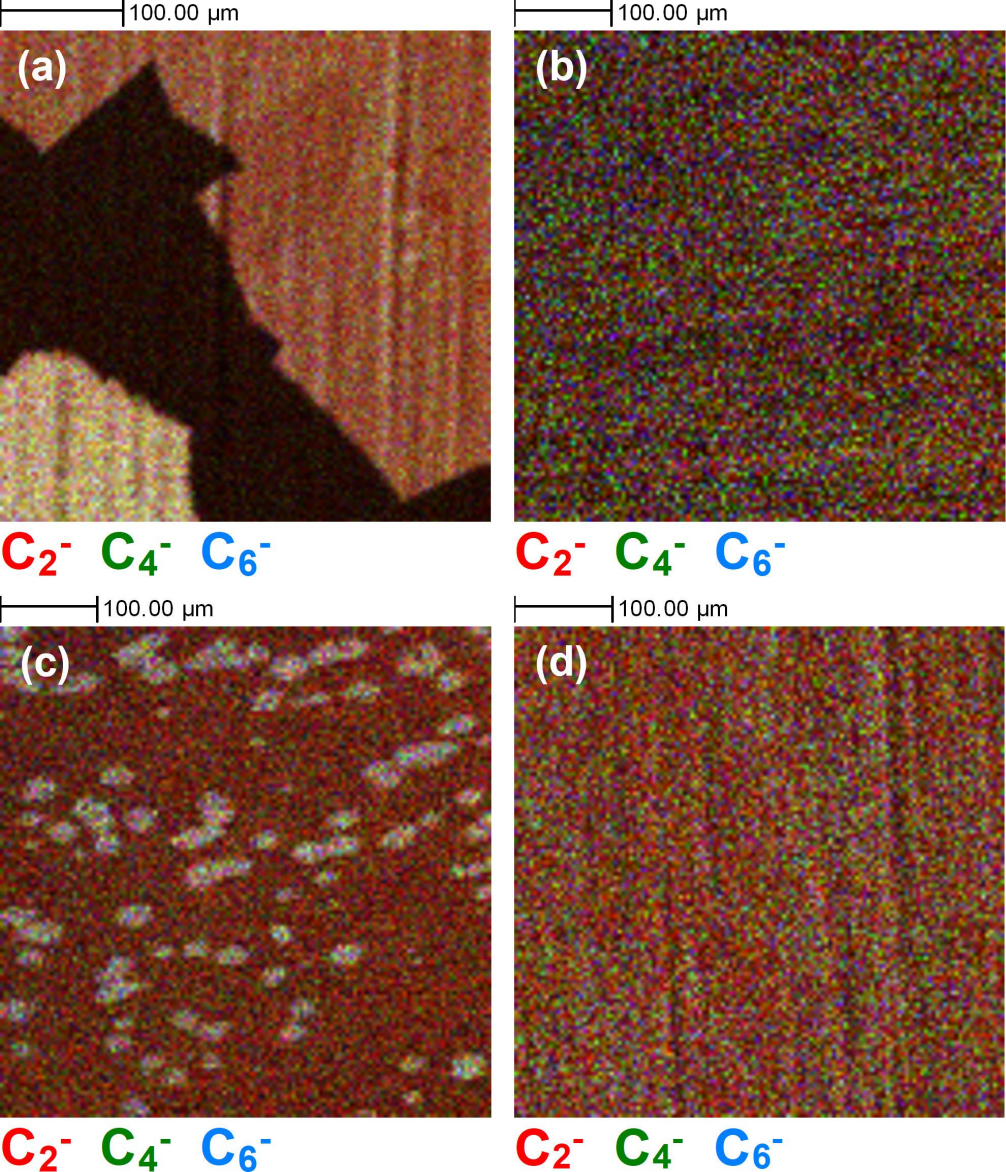
Representative ToF-SIMS surface images (500 µm × 500 µm) of C_2_^−^ (red), C_4_^−^ (green), and C_6_^−^ (blue) ion signals, after different graphene growth experiments, that is, (a) back side oxidation + Ar annealed (BO), (b) Ar:H_2_ annealed (Ar:H_2_), (c) electro-polished + Ar:H_2_ annealed (EP), and (d) Ar annealed (Ar) samples.

ToF-SIMS imaging of graphene grown on Cu foils after various surface pre-treatments, as shown in Figure 1, reveals dramatic differences in the nucleation density of graphene. Using the negative secondary ion signal from polyatomic carbon ions (C*_n_*^−^), which are indicative of graphene, coming from fragmentation of the graphene carbon lattice [28], we can easily observe the areas of graphene coverage on the copper foil surfaces. Depending on the pre-treatment, the graphene nucleation density varies significantly, resulting in variations in coverage. For the BO and EP samples in Figure 1a,c, nucleation is sufficiently low to observe individual nucleation sites. In the case of the BO sample, these could be on the scale of millimetres, whereas, with the Ar and Ar:H_2_ samples in Figure 1b,d, there is complete coverage of the samples with carbon within the measured area. Images particularly dominated by the C_2_^−^ signal (red) in certain regions have a variation in the surface chemistry not necessarily related to graphene. Interestingly, there is a C_2_^−^ signal observed relatively uniformly across the surface of the EP sample in Figure 1c, despite the low nucleation density of graphene on this surface.

This is distinctly different from the BO sample, which similarly had a low nucleation density and growth rate, but a relatively lower C_2_^−^ ion signal outside of the graphene regions. This is consistent with previous reports that showed the scavenging effect of oxygen diffused within Cu after the oxidation process. Carbon present in the Cu foil is redistributed [43] with the removal of contamination effectively enhancing the relative C_2_^−^ signal contribution from graphene [46]. With the electro-polishing process, the excessive carbon at the surface of the foil and from protrusions of carbon along rolling striations is significantly removed; however, some of these areas of high carbon concentration still exist within the foil, leading to a greater nucleation density than for the BO sample, as previously reported (≈1 × 10^2^ mm^−2^ vs ≈1 × 10^−2^ mm^−2^) [43]. This is still greatly reduced when compared to the Ar and Ar:H_2_ samples. Including higher-order carbon ion peaks (C_2+_*_n_*^−^) in Figure 1 helps to distinguish between the areas consisting primarily of carbon contamination in the Cu foil and the graphene areas, as the latter have a higher relative contribution of C_4_^−^ and C_6_^−^ polyatomic carbon species in the mass spectra [46]. This is clear for the BO sample in Figure 1a and the EP sample in Figure 1c, which still have C_2_^−^ signals across all of the imaged areas. However, it is still more difficult to distinguish carbon contamination from graphene for the Ar and Ar:H_2_ samples in Figure 1b,d due to the increased graphene coverage on these samples as a result of the greater number of graphene nucleation sites. These lead to a greater graphene edge contribution, which in turn leads to greater fragmentation potential and smaller carbon ion clusters detected in the ToF-SIMS measurements. The inherent C_2_^−^ signal originating from the foil and from ambient surface contamination, which we would expect at the same level on all samples, also contributes to this difficulty.

To confirm the presence of a protective graphene layer on the surface of the Cu foils, as opposed to just carbon contamination, we can look for evidence of the substrate Cu signal, specifically the signal related to Cu oxide. The gas permeation barrier properties of graphene are well known [47–48], and as such it is possible to compare the level of oxidation of the Cu foil after graphene growth by comparing the regions where the Cu surface is exposed and where graphene is present. As mentioned previously, all samples had been exposed to atmosphere for longer than four weeks prior to measurements.

**Figure 2:**
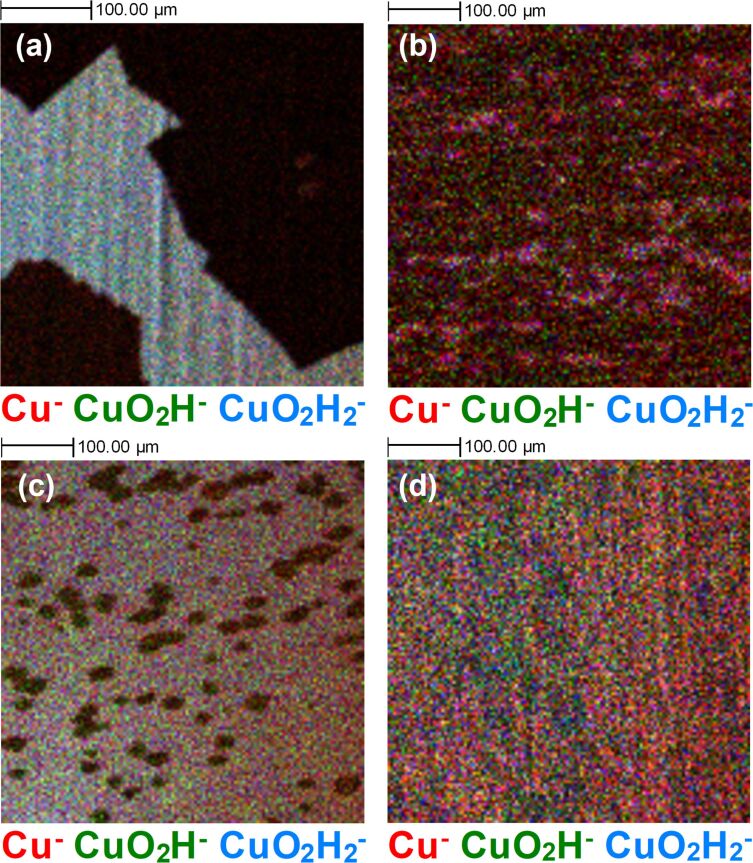
ToF-SIMS surface images (500 µm × 500 µm) of substrate-related Cu^−^ (red), CuH_2_O_2_^−^ (green) and Cu_2_H_3_O_2_^−^ (blue) ion signals after different graphene growth experiments, that is, (a) back side oxidation + Ar annealed (BO), (b) Ar:H_2_ annealed (Ar:H_2_), (c) electro-polished + Ar:H_2_ annealed (EP), and (d) Ar annealed (Ar) samples, for the same areas of the samples as in Figure 1.

Figure 2 shows ToF-SIMS surface images of the same four regions shown in Figure 1, this time representing the Cu^−^, CuO_2_H^−^, and Cu_2_H_3_O_2_^−^ ion signals. There are clear differences observed for the four samples in terms of oxidation of the underlying substrate. For the BO sample, as expected, there is no detectible Cu oxide in the regions where a graphene signal has been identified. Similarly, the EP sample shows clear distinction between oxidised Cu and graphene regions. The Ar:H_2_ and Ar samples in Figure 2b and Figure 2d, respectively, exhibit contrasting results. For the Ar:H_2_ annealed sample in Figure 2b, there are clear streaks of oxide-related signals, despite the presence of a C_2_^−^ signal across the entirety of the surface in Figure 1b suggesting incomplete graphene coverage, and other carbon-containing species contributing to the coverage. Similarly, for the Ar sample in Figure 2d, despite a C_2_^−^ ion signal being detected relatively uniformly covering the measured area of the surface in Figure 1d, we still detect an ion signal related to Cu oxide across the imaged area, but without the same evidence of streaks of oxide as seen for the Ar:H_2_ sample. The suggests the extent of oxidation of the copper does vary, with regions with greater Cu^−^ ion signal consistent with less oxidation. This could suggest variations in the level of defects present within the graphene films, which facilitate oxygen diffusion, depending on the growth process. The variation in the colour distribution also reflects potential variations in the composition of the oxide layer that forms on the surface.

**Figure 3:**
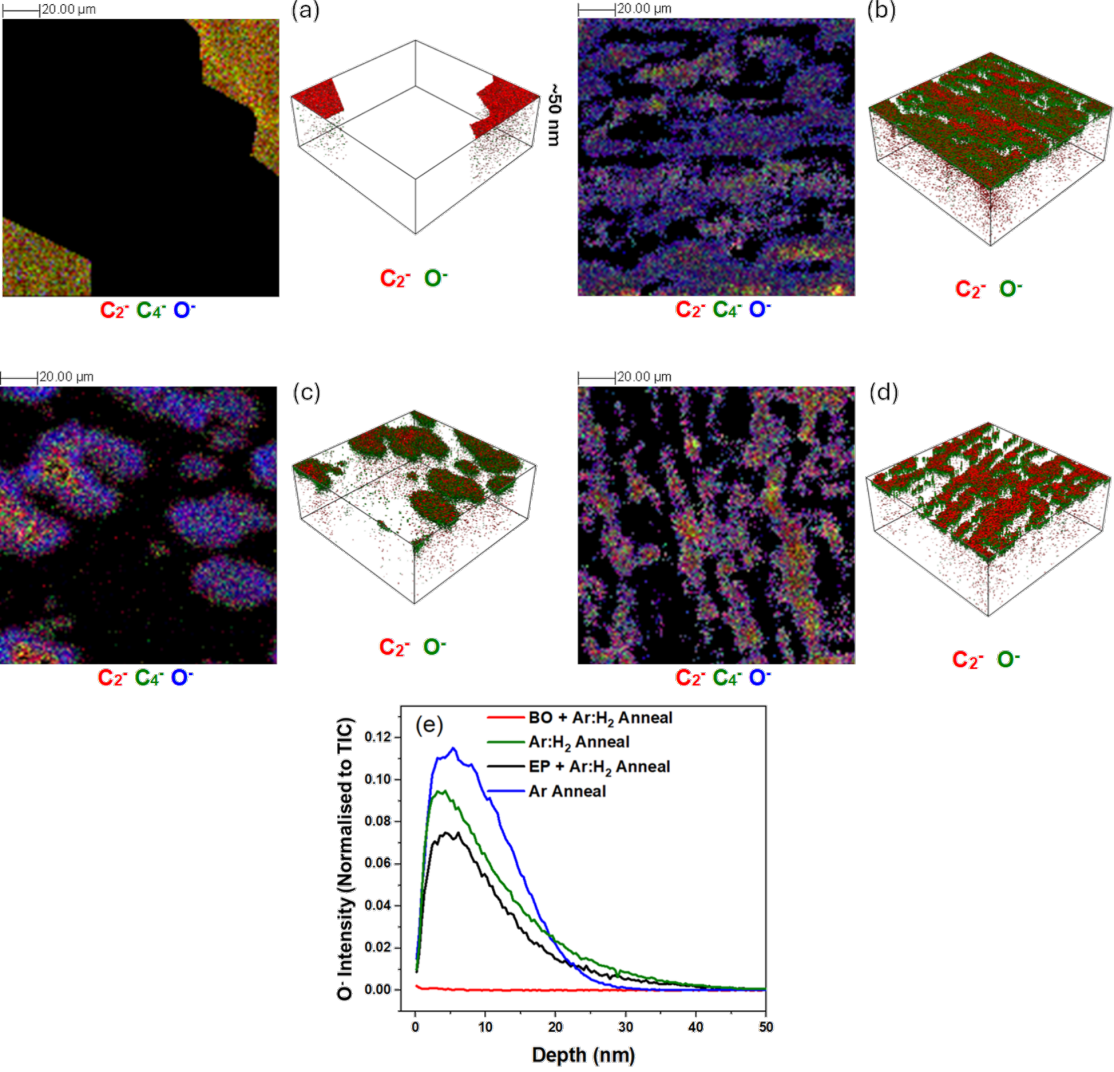
ToF-SIMS depth profile images of graphene grown on copper foils utilising different graphene growth mechanisms from the first 50 nm of the sample, for (a) back side oxidation + Ar annealed (BO), (b) Ar:H_2_ annealed (Ar:H_2_), (c) electro-polished + Ar:H_2_ annealed (EP), and (d) Ar annealed (Ar) samples, over an imaged area of 150 µm × 150 µm. The images are reconstructed to only show areas where graphene-related ion signals were detected, with the C_2_^−^ (red) indicative of graphene and the O^−^ (blue) indicative of oxygen (black indicates no ion signal shown). Figure 3e shows the depth profile for the O^−^ ion signal from the same regions where the C_2_^−^ signal was detected for the four samples.

To further explore the extent of the substrate oxidation in the presence of graphene, 3D ToF-SIMS imaging was performed to determine oxygen permeation in areas where graphene material was present on the surface of the Cu foils. A region of 400 µm × 400 µm was sputtered with a 10 keV Cs ion beam, with concurrent ToF-SIMS images collected from a 150 µm × 150 µm region in the centre of the sputtered crater, at regular intervals of time during the sputter process up to a depth of ≈180 nm. To compare the extent of oxygen diffusion through the graphene layers, regions of interest were selected in the images corresponding to the graphene signal (i.e., regions where multiple polyatomic carbon ion (C*_n_*^−^) signals were observed). For the Ar and Ar:H_2_ samples, this comprised the majority of the imaged area, consistent with Figure 1 where large area coverage was observed. All other regions in the images were excluded, and the resultant data was reconstructed for the total sputtered depth as shown in Figure 3, with all images from the first ≈50 nm of the profile combined into a single image on the left, and the corresponding 3D image on the right. Regions where there was no obvious graphene-related signal were excluded from the reconstructions for clarity and are seen in the image as black.

In the case of the BO sample, where we observed clear evidence of individual graphene domains (>100 µm) on the surface in Figure 1a, there is virtually no observable oxygen signal (O^−^) detected under the graphene layer, with only trace amounts related to surface contamination from ambient exposure detected on the surface. This is of note as the graphene/Cu interaction strength is known to depend on the Cu crystal orientation as well as the epitaxial relationship [49–51], which leads to anisotropy in the Cu oxidation rate at the graphene/Cu interface for different Cu orientations. Thus, the oxidation of Cu underneath graphene strongly depends on the Cu facet [51–54], and it is likely that in this case, due to the size of the graphene domains, it can span different Cu facets. Yet, no oxidation is observed.

For the other three samples, however, an obvious oxygen signal is detected. This O^−^ signal is observed in areas underneath the graphene as well as at elevated levels at the edges of some of the graphene domains, seen most clearly in the EP sample in Figure 3c. Where there is a high nucleation density and large area coverage, as in the case of Figure 3b,d, it might be expected that the presence of a greater number of grain boundaries between individual graphene domains could facilitate oxygen diffusion and subsequent substrate oxidation more readily at these locations [41,55–56]. The oxygen source could be from ambient exposure, residual water vapour in the CVD reactor, or outdiffusion of oxygen remaining in the Cu foil after growth. However, in the case of the latter, we would expect to see oxygen under the graphene in the BO sample due to the intentional addition of oxygen in this sample, which is not observed. This would suggest a post-growth ambient route as the main mechanism for oxidation of the Cu, where oxygen is penetrating through/underneath the graphene.

The corresponding depth profile plots for the four samples are shown in Figure 3e. The extent of oxygen permeation under the graphene is similar for the three samples where oxygen was detected, which results in a self-limited oxide thickness of ≈5 nm, consistent with previous reports [57]. In the case of the EP sample in Figure 3c, where the graphene domains are known to be single-crystalline in nature (without grain boundaries), an explanation is needed to rationalise the presence of oxygen underneath the graphene and why we do not observe it for the BO sample. Previously, a detailed understanding of the carbon contamination in the Cu foil was able to help explain the reduced nucleation density observed for the BO sample [43]; thus, looking at the other contamination species present on the surface could help to provide an explanation. Due to the high temperatures during growth and the relatively high purity of the source gases and materials, the naive expectation is that the chemistry of the graphene surface should be very straightforward, with only carbon being present on top of copper or copper oxide. However, as recent studies have shown through energy-dispersive X-ray spectroscopy mapping [30], there are significant other contaminants detectable on high-purity Cu foils that can influence graphene nucleation and can remain after growth [31]. A more detailed examination of what chemical species coexistent with the graphene on the surface of the Cu foils is necessary.

**Figure 4:**
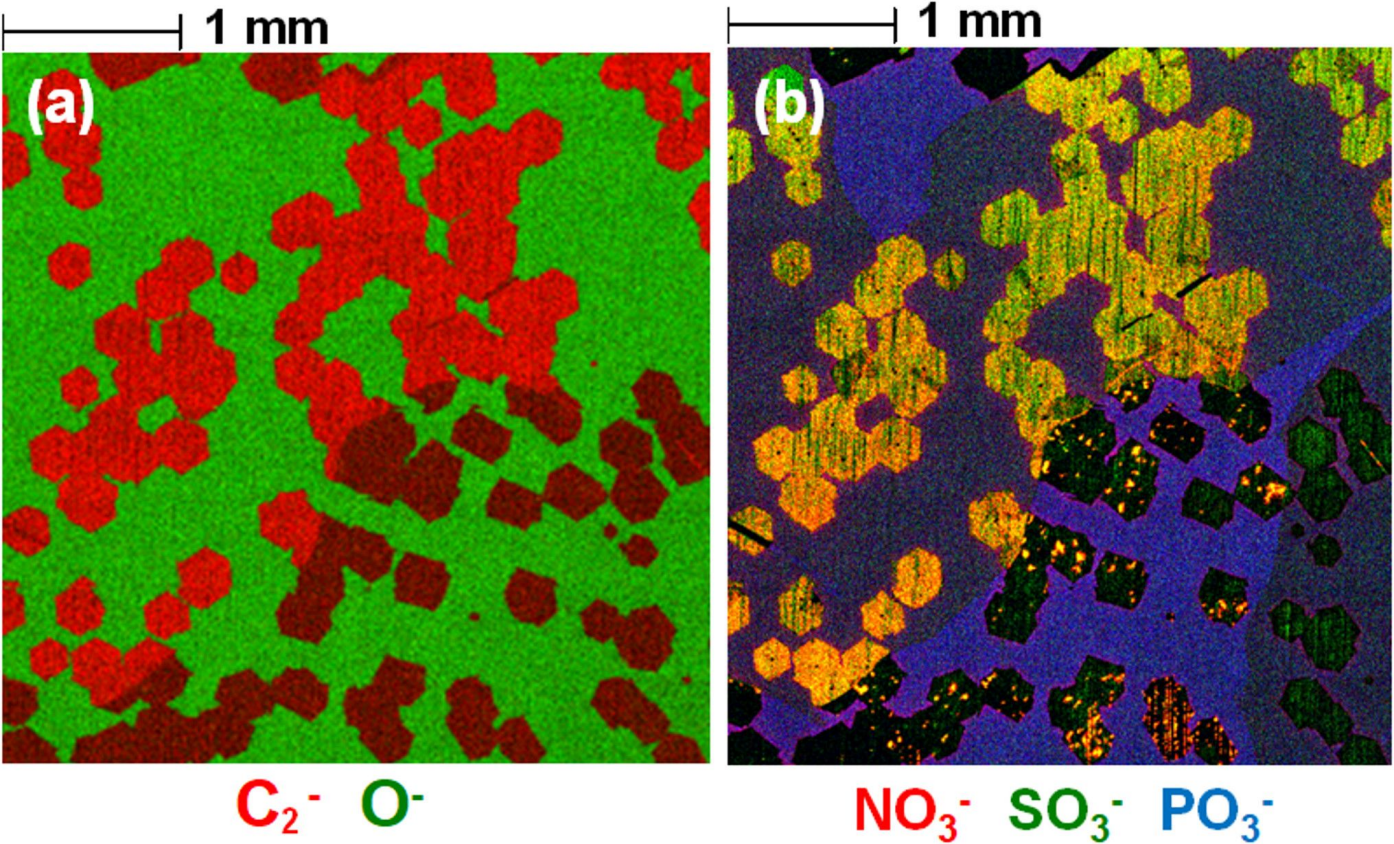
ToF-SIMS images (4 mm x 4 mm) from the surface of the BO sample, showing in (a) the C_2_^−^ graphene-related ion signal in red and the O^−^ ion signal from the oxidised copper surface in green. The presence of contaminations on the surface of graphene is shown in (b) with nitrate, sulphite, and phosphite species detected in various locations and in some instances strongly correlated with each other.

Figure 4 shows a 4 mm × 4 mm secondary ion image of the BO sample surface. In Figure 4a, we can see the contrast between the carbon-rich graphene regions in red and the oxidised Cu substrate in green. If we look at the image in Figure 4b, which overlays signals related to nitrate (red), sulphate (green), and phosphate (blue), we can see that there is a much more diverse chemistry present than just graphene.

Although there is no obvious correlation between the presence of any of these detected species on the graphene nucleation process itself (other than previously reported carbon contamination in the Cu foil) [43], it is reasonable to assume that a non-uniform distribution of chemical species is not desirable when consistency of a material will be important for uptake of graphene in any future industry application. It also appears that there are variations in the presence and distribution of these species, depending on the grain orientation of the underlying copper foil; the grain structure evident in Figure 4a as a change in intensity of the C_2_^−^ signal due to crystal orientation enhancement of the ion signal. This can further add to the potential variation in chemical composition of a grown graphene layer on copper foils, where crystal orientation is not well controlled [54].

**Figure 5:**
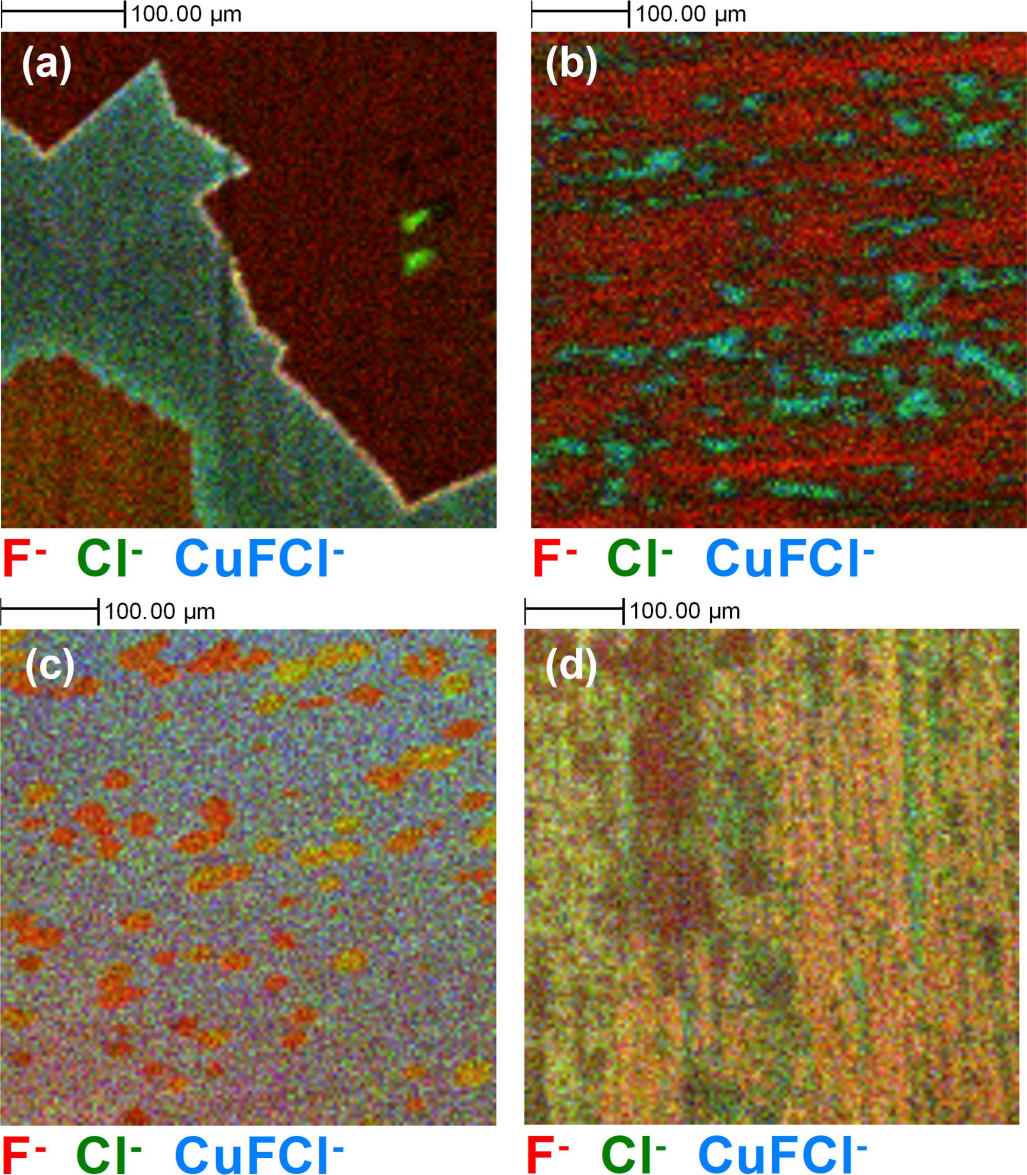
ToF-SIMS surface images (500 µm × 500 µm) of the F^−^ (red), Cl^−^ (green), and CuFCl^−^ (blue) ion signals after graphene growth on copper with different growth mechanisms, from (a) back side oxidation + Ar annealed (BO), (b) Ar:H_2_ annealed (Ar:H_2_), (c) electro-polished + Ar:H_2_ annealed (EP), and (d) Ar annealed (Ar) samples for the same areas of the samples as in Figure 1 and Figure 2.

To explore some of these species in more detail, Figure 5 shows 500 µm × 500 µm 2D ToF-SIMS surface images of the same areas of the four samples as in Figure 1 and Figure 2, with the F^−^ (red), Cl^−^ (green), and CuFCl^−^ (blue) signals overlaid. This CuFCl^−^ ion signal is not necessarily indicative of a chemical bond of this nature, but would be related to copper, chlorine, and fluorine atoms being present in close proximity in the sample. In all cases, we see evidence of a fluorine signal co-located with the graphene-related signal from Figure 1. There is also a chlorine signal observed on all the samples, primarily in the region of the exposed substrate, evident in particular in Figure 5a,b for the BO and Ar:H_2_ samples. However, on the EP and Ar samples in Figure 5c,d, there is a chlorine signal in areas where there is also a fluorine signal (with colour mixing this is revealed as a yellow signal). Also, for the BO sample in Figure 5a we observe an elevated fluorine and chlorine signal at the immediate edge of some of the graphene domains.

**Figure 6:**
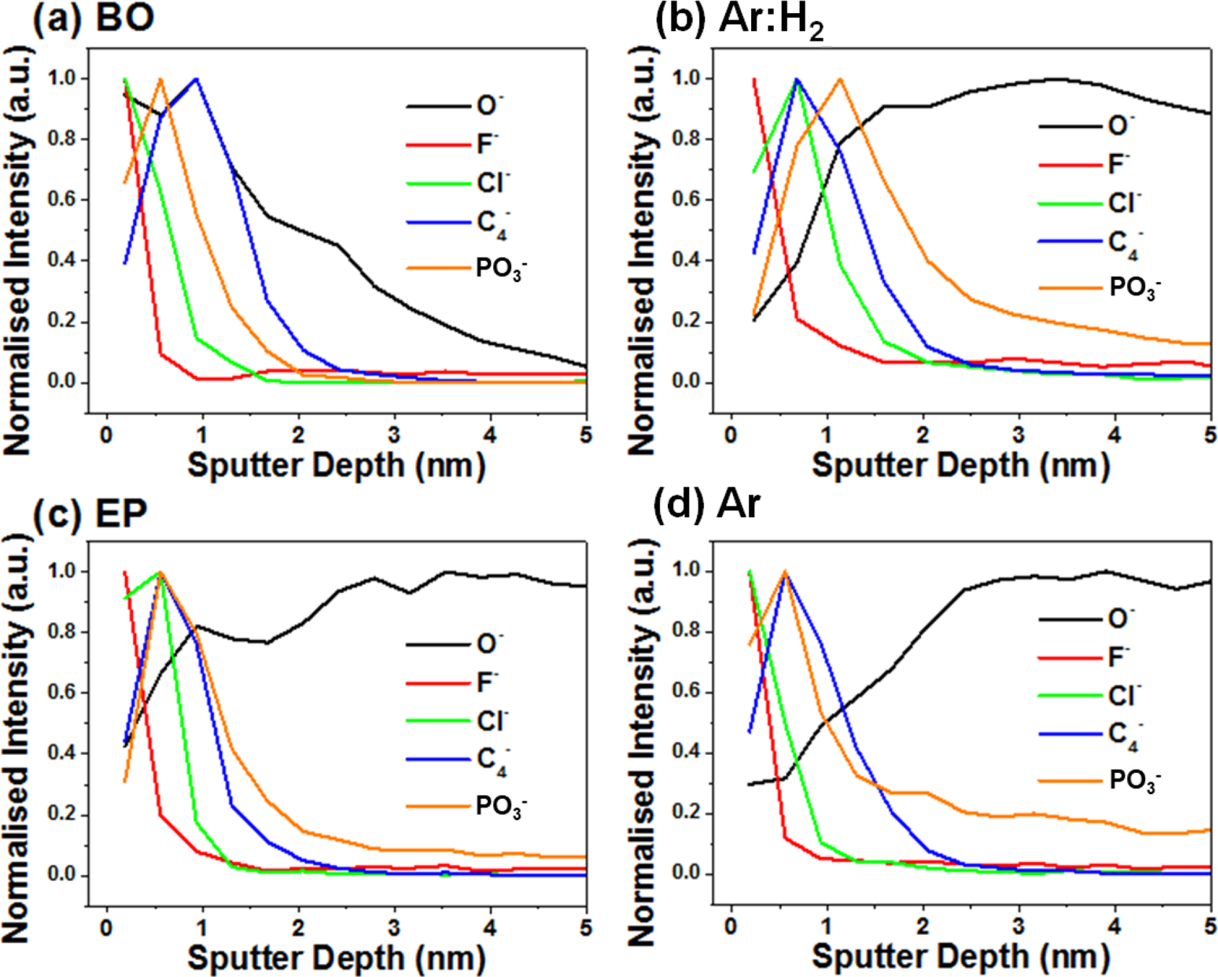
ToF-SIMS depth profiles from (a) back side oxidation + Ar annealed (BO), (b) Ar:H_2_ annealed (Ar:H_2_), (c) electro-polished + Ar:H_2_ annealed (EP), and (d) Ar annealed (Ar) samples, showing the distribution of chemical species within the first 5 nm of the surface.

This suggests that variations in the processing conditions result in variations in the distribution of these chemical species on graphene. We can look for these species at the near-surface region of the graphene-covered areas of the samples during depth profiling in Figure 6 (using the same graphene-covered areas from Figure 3). This reveals that the F^−^ and Cl^−^ signals appear primarily at the surface of the sample. The PO_3_^−^ signal appears in all samples but is only observed to decrease to the background noise level in the BO sample and remains detectable in the other three samples over the depth shown here, and to greater depth (>50 nm). Phosphorous is a common additive to copper metals during the smelting process, to aid removal of oxygen and improve the copper material performance, in terms of strength, ductility, and corrosion resistance, although there can be a trade-off in terms of reduced conductivity [58]; while typically not intentionally added to copper used for CVD purposes, it can still be present as a contaminant from the copper production process.

**Figure 7:**
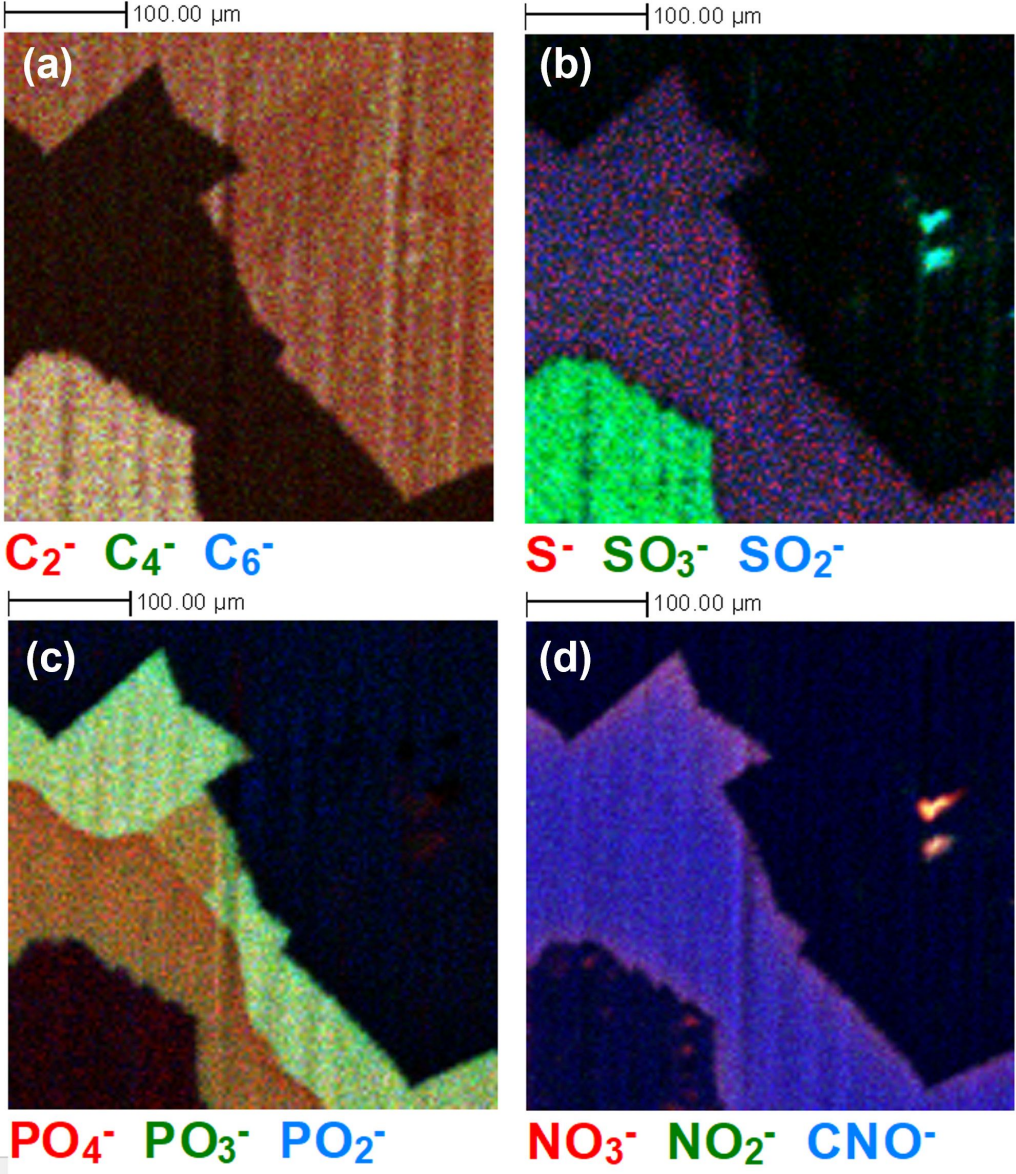
ToF-SIMS surface images (500 µm × 500 µm) from the BO sample showing (a) C*_n_*^−^ species related to graphene, (b) sulphur-containing species, (c) phosphorous-containing species and (d) nitrogen-containing species, indicating the significant variation in surface chemistry.

The 4 mm × 4 mm ToF-SIMS image of the BO sample in Figure 4b also reveals the presence of sulphite, nitrate, and phosphite species on the surface of the sample, with the immediate surface localisation of these confirmed by the depth profiles. Figure 7 explores the variation in the composition of these species in more detail for the BO sample, with Figure 7a corresponding to the same region shown in Figure 1a. The sulphur ion signals in Figure 7b indicates that there is a location specific variation in the chemistry of the species present on the regions where graphene is present, with SO_3_^−^ clearly observed in the area of the graphene domain in the lower left of the image, but no obvious signal from this species detected from the domain in the upper right of the image.

**Figure 8:**
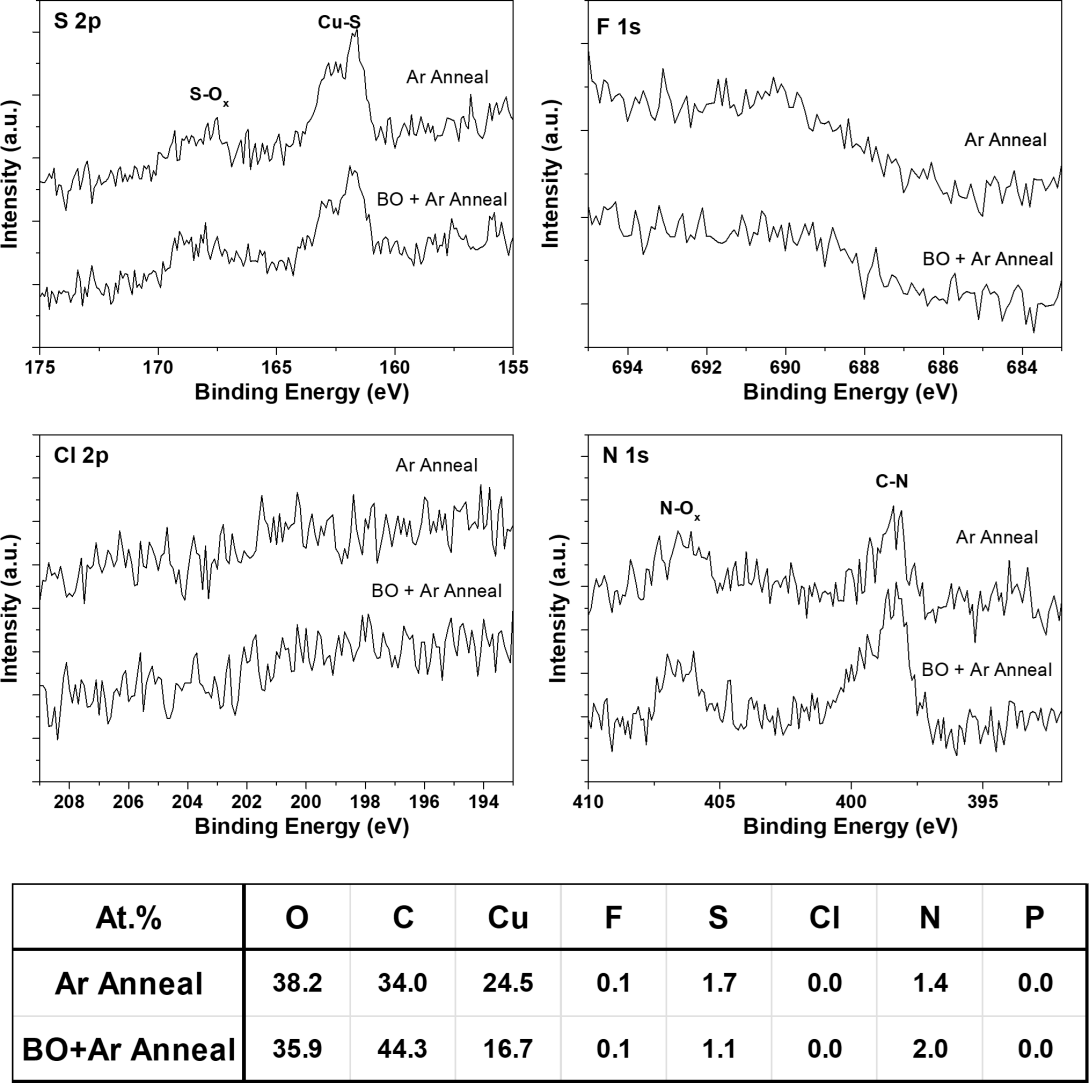
XPS spectra from the Ar annealed, and back side oxidation + Ar annealed samples prior to graphene growth comparing the level of contamination species present on the surface of the copper foils from the S 2p, F 1s, Cl 2p, and N 1s core level spectra. The calculated concentration values based on averaging of values from two different areas on the same samples are also shown.

While ToF-SIMS is very sensitive to trace amounts of material, actual quantification of the amount present is difficult without having reliable reference materials, with minimal topography and consistent crystal or grain orientation within the samples. XPS, while not having the same sensitivity, is a very useful technique for determining elemental composition to the level of >0.1% concentration within the first 10 nm of the sample. Figure 8 shows the XPS spectra from Ar and BO samples prior to graphene growth, to compare the level of contaminant species present on the surface of the foils without the spectra being dominated by signal from the graphene.

Sulphur and nitrogen species are detected quite readily at concentration levels between 1% and 2 % of the total surface composition. Interestingly, the signal detected from the F 1s core level was very close to the detection limits of XPS for both samples, and it was not possible to resolve a signal from the P 2p core level spectra, despite F- and P-containing ion species being easily detected by ToF-SIMS from the same samples, highlighting the enhanced sensitivity of ToF-SIMS.

**Figure 9:**
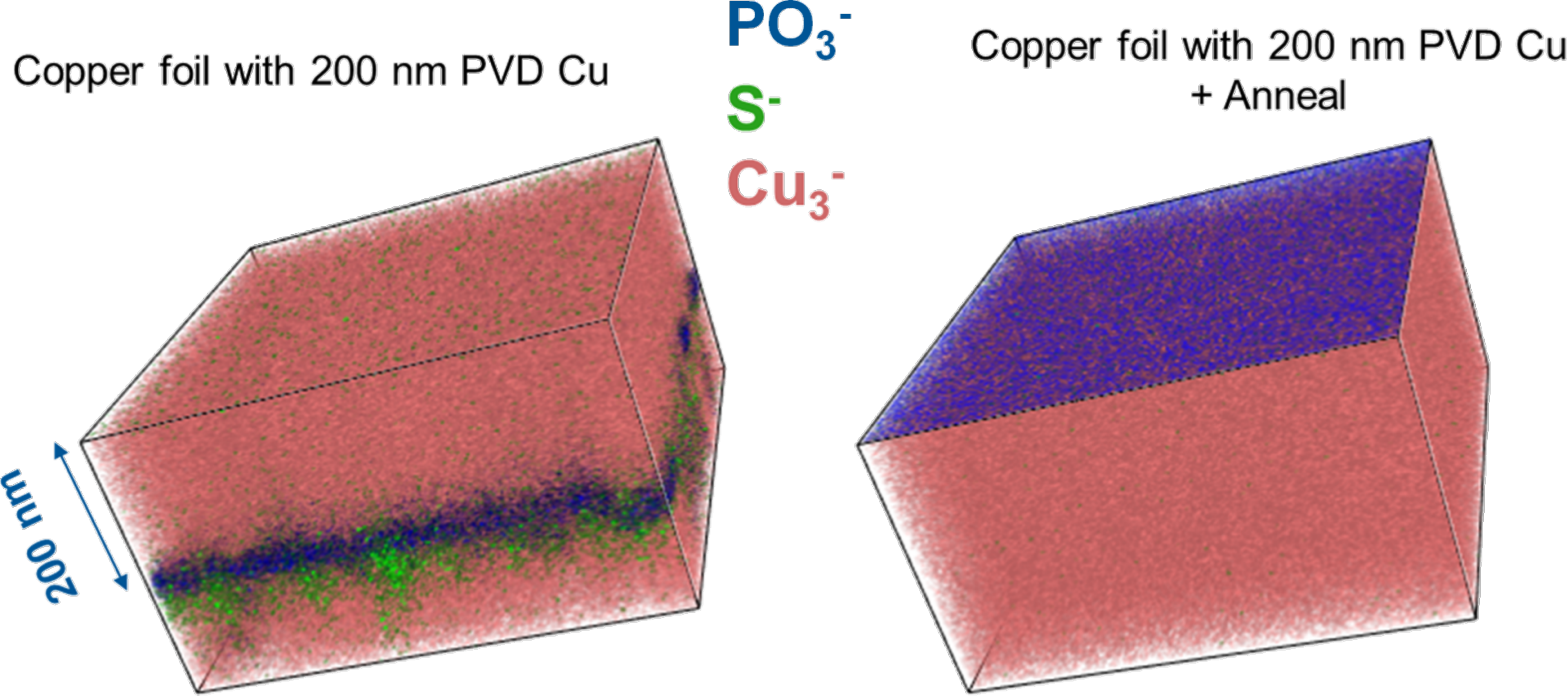
3D ToF-SIMS images (150 µm × 150 µm) of a 200 nm PVD high-purity copper layer deposited on top of a copper foil (left) and after annealing at the graphene growth temperature (right), indicating the migration of P within the copper at elevated temperatures.

To confirm that P was coming from the Cu foil, a 200 nm layer of high-purity Cu was deposited on top of a sample of the same Cu foil used for graphene growth by physical vapour deposition (PVD). ToF-SIMS depth profile measurements were taken before and after annealing to the graphene growth temperature. The corresponding 3D ToF-SIMS images are shown in Figure 9. For the as-deposited sample, there is no evidence of phosphorous or sulphur signals present on the surface of the sample; however, upon depth profiling through the 200 nm PVD layer, we observe the presence of PO_3_^−^ and S^−^ signals at the interface with the Cu foil substrate. Upon annealing, the interfacial PO_3_^−^ signal is observed to diffuse to the surface of the PVD layer, indicating that the phosphorous species are very mobile within the Cu. The sulphur signal that was previously observed is no longer detectable, indicating that the corresponding sulphur compound is likely to be volatile under the annealing conditions. This all suggests that it is unlikely possible to prevent P buildup at the surface or interface between graphene and Cu foils, where P is present within the foils prior to growth, due to the apparent stability of these species at the surface of the foil.

This facile migration of phosphorous species to the surface of Cu [59] would suggest that, in the case of the BO sample, it is not favourable for it to remain at the interface between the graphene and the Cu foil as minimal PO_3_^−^ was observed collocated with the graphene in the ToF-SIMS images in Figure 7c and during depth profiling. It is possible that, in this case, the van der Waals forces at the interface between graphene and copper may be sufficient to squeeze out any species that appears towards the edges of the graphene [60]. This would explain the elevated F^−^, Cl^−^, and nitrate-related ion signal intensities at these graphene edge locations as observed in Figure 5a and Figure 7d. The Cu surface orientation, which will vary across the surface of the foil, could also play a role in the chemical variations, as evidenced by the differences in the location of sulphate-related species in Supporting Information File 1, Figure S1. The transfer of some of these contamination species along with the graphene layer to any other substrate of interest is highly probable, with previous ToF-SIMS studies of transferred CVD graphene from Cu indicating the presence of substrate contamination-related signals after transfer [27], which has implications for the subsequent properties of the graphene layer.

## Conclusion

Graphene growth on Cu foils under various processing conditions was probed by ToF-SIMS analysis. It showed significant variations in the chemistry at the surface of the Cu foils. Chemical species containing chlorine, fluorine, nitrogen, and sulphur are readily detected due to the high sensitivity of ToF-SIMS, with evidence that these species originate, at least to some degree, from the Cu foil despite the relatively high purity of the material. The ability of these materials to migrate to the surface of the Cu foil at graphene growth temperatures is demonstrated. This has implications for applications using CVD graphene where the presence of different chemical species could impact on the intended application properties, such as electronic devices or sensors. Furthermore, the variation in the coverage of chemical species must be addressed for graphene to be reliably used in industry applications. This study highlights the need to further understand the chemical species present during graphene growth to optimise growth processes and to minimise variation in chemical composition. Also, it reveals the need for careful consideration and further studies of the copper foils used in the growth process, as well as contamination mitigation strategies to optimise the graphene produced for real-world applications.

## Supporting Information

File 1 Additional figures.

## Data Availability

Data generated and analyzed during this study is available from the corresponding author upon reasonable request.
