# Supplementary material for: Time of flight secondary ion mass spectrometry imaging of contaminant species in chemical vapour deposited graphene on copper
Source: Beilstein J Nanotechnol. 2026 Jan 21;17:200–13. doi: 10.3762/bjnano.17.13 (PMC12834054; doi:10.3762/bjnano.17.13)
Supplement: File 1 — Additional figures. [file Beilstein_J_Nanotechnol-17-200-s001.pdf]

BEILSTEIN JOURNAL OF NANOTECHNOLOGY

Supporting Information

for

Time of flight secondary ion mass spectrometry imaging of contaminant species in chemical vapour deposited graphene on copper

Barry Brennan, Vlad-Petru Veigang-Radulescu, Philipp Braeuninger-Weimer, Stephan Hofmann and Andrew J. Pollard

Beilstein J. Nanotechnol. **2026,** *17,* 200–213. doi:10.3762/bjnano.17.13

Additional figures

License and Terms: This is a supporting information file under the terms of the Creative Commons Attribution License (https://creativecommons.org/licenses/by/4.0). Please note that the reuse, redistribution and reproduction in particular requires that the author(s) and source are credited and that individual graphics may be subject to special legal provisions.
The license is subject to the *Beilstein Journal of Nanotechnology* terms and conditions: (https://www.beilstein-journals.org/bjnano/terms)

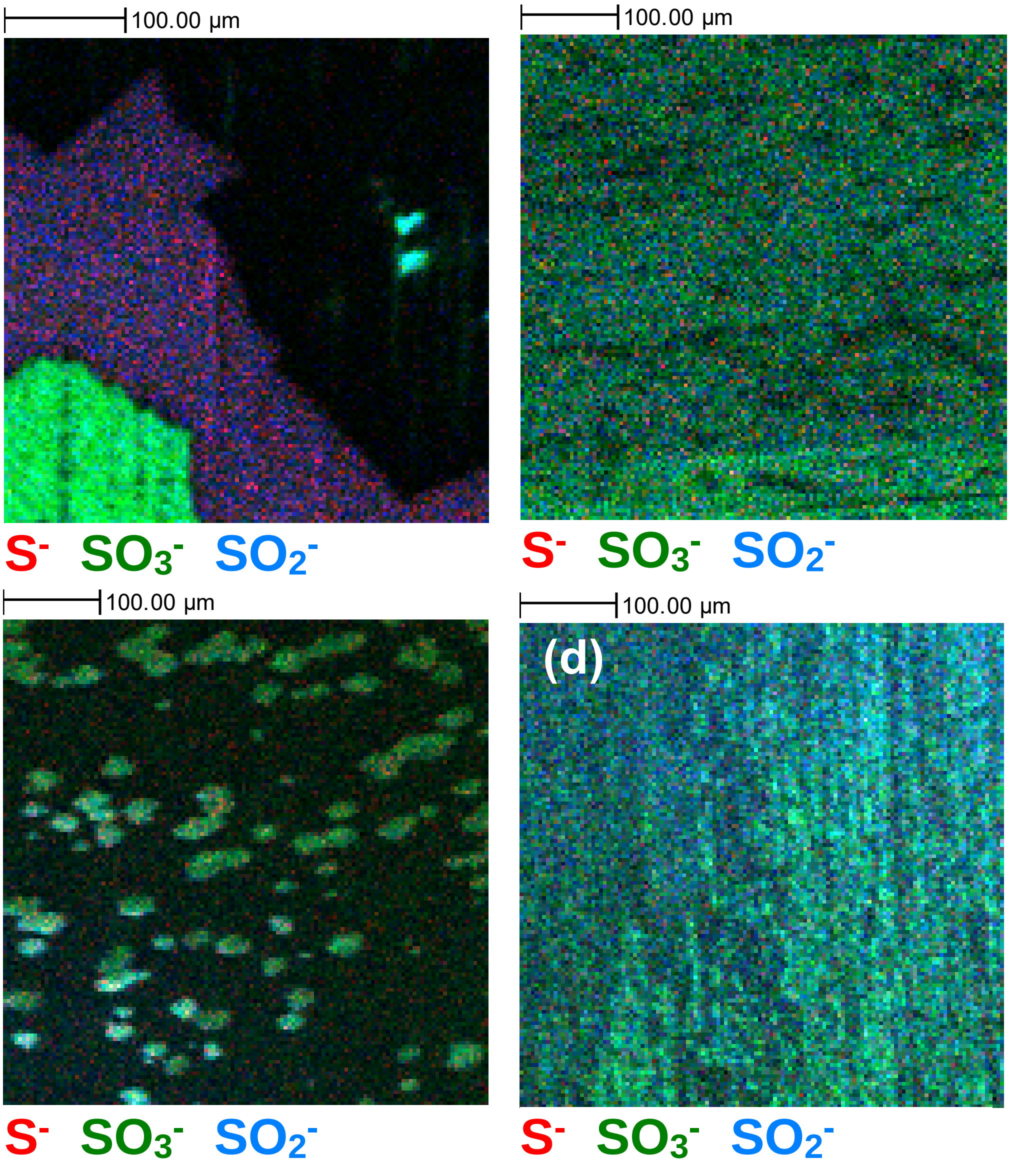

Figure S1: ToF-SIMS images (500 µm × 500 µm) of the S^- (red), SO_3^- (green) and SO_2^- (blue) ion signals after graphene growth on copper with different growth mechanisms, from (a) backside oxidation + Ar annealed (BO), (b) Ar:H_2 annealed(Ar:H_2), (c) electro-polished + Ar:H_2 anneal (EP), and (d) Ar annealed (Ar) samples, for the same areas of the samples as in Figure 1 and 2.

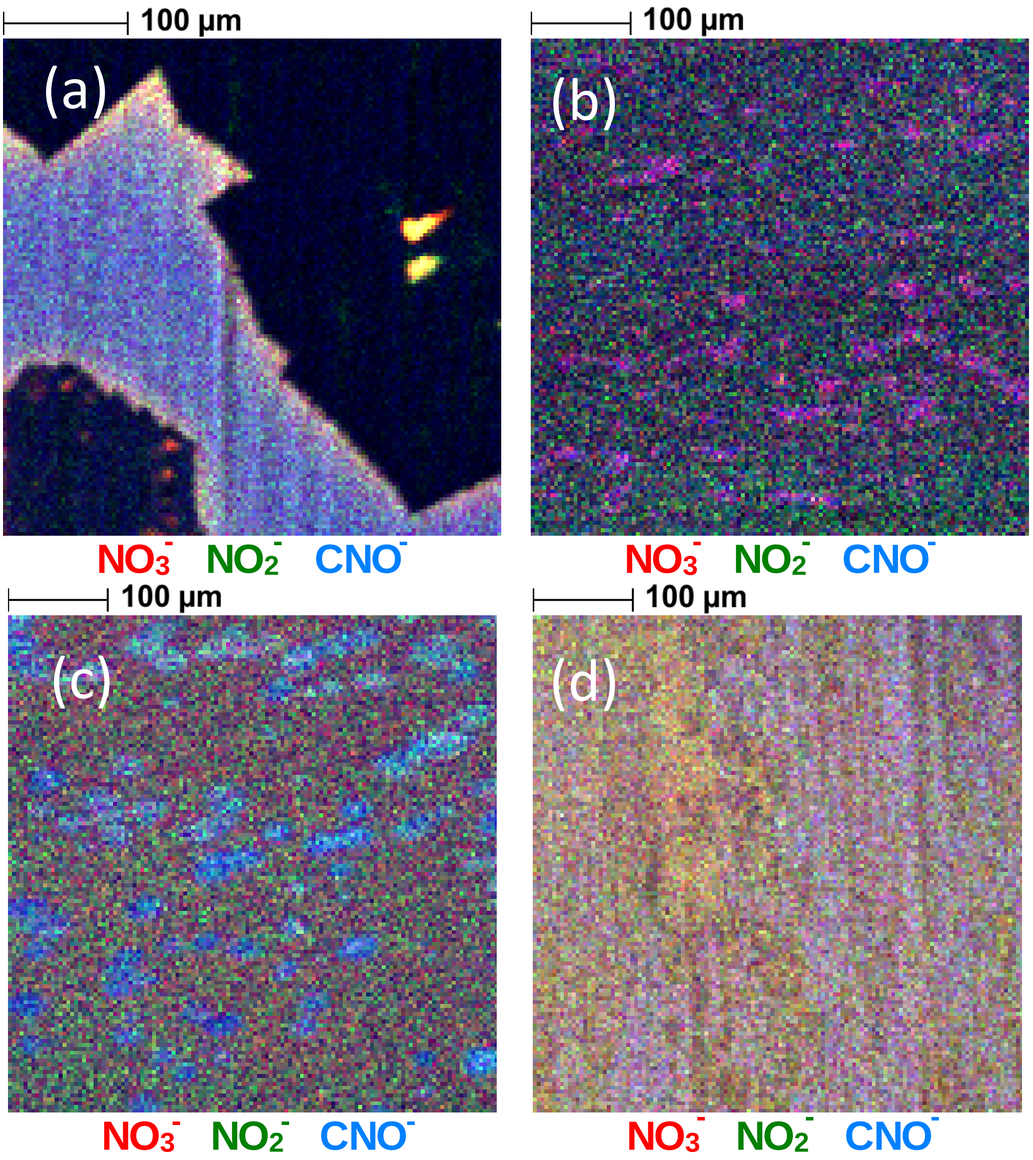

Figure S2: ToF-SIMS images (500 µm × 500 µm) of the NO_3^- (red), NO_2^- (green) and CNO^- (blue) ion signals after graphene growth on copper with different growth mechanisms, from (a) backside oxidation + Ar annealed (BO), (b) Ar:H_2 annealed(Ar:H_2), (c) electro-polished + Ar:H_2 anneal (EP), and (d) Ar annealed (Ar) samples, for the same areas of the samples as in Figure 1 and 2.

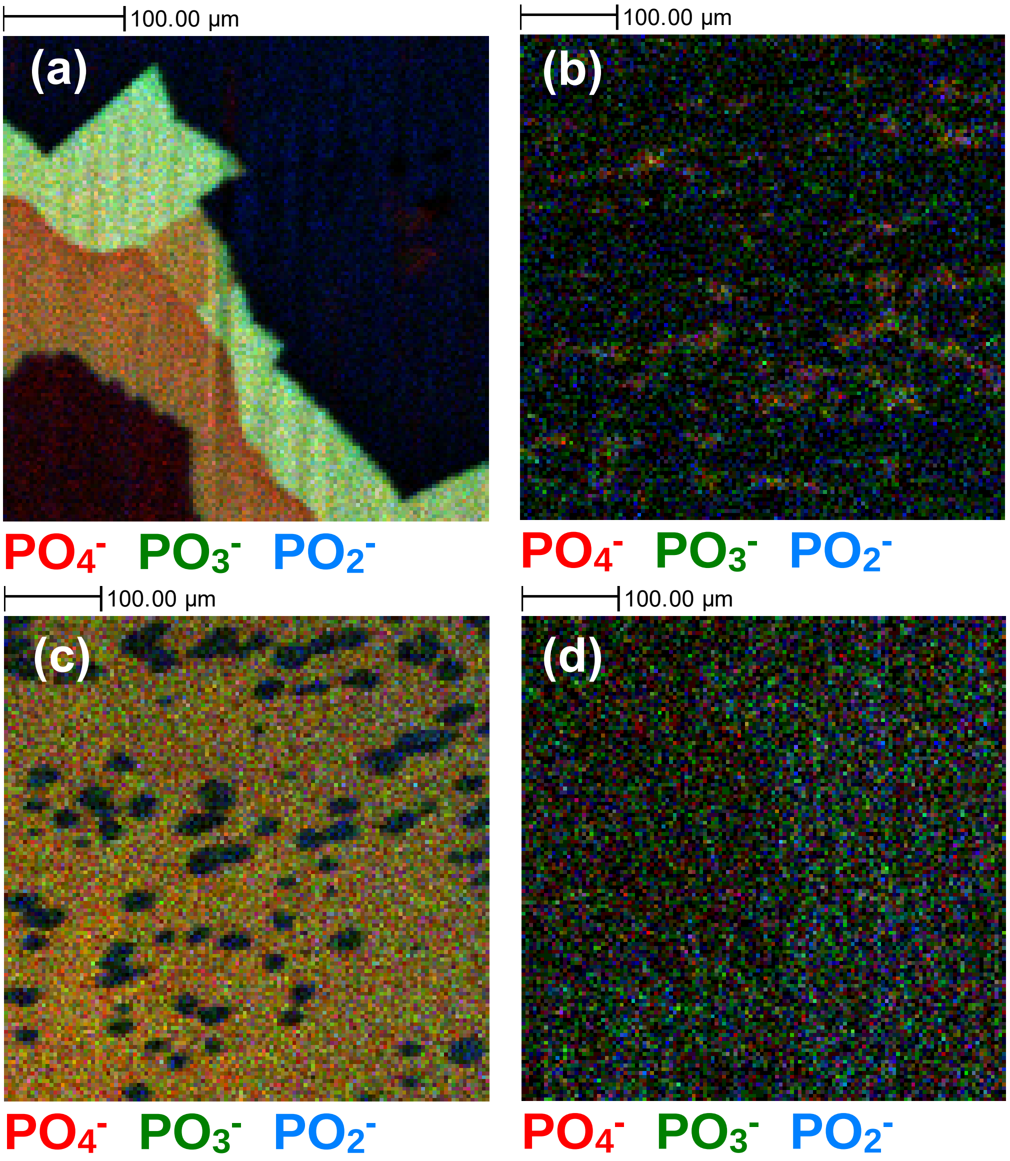

Figure S3: ToF-SIMS images (500 µm × 500 µm) of the PO_4^- (red), PO_3^- (green) and PO_2^- (blue) ion signals after graphene growth on copper with different growth mechanisms, from (a) backside oxidation + Ar annealed (BO), (b) Ar:H_2 annealed(Ar:H_2), (c) electro-polished + Ar:H_2 anneal (EP), and (d) Ar annealed (Ar) samples, for the same areas of the samples as in Figure 1 and 2.
